# METTL3 Silencing Suppresses Cardiac Fibrosis Post Myocardial Infarction via m6A Modification of SMOC2

**DOI:** 10.1111/jcmm.70829

**Published:** 2025-09-05

**Authors:** Yanru He, Xiaodong Pan, Zhuyuan Liu, Pengfei Zuo, Zulong Sheng, Chunshu Hao, Zaixiao Tao, Zhongpu Chen, Jiali Song, Genshan Ma, Sunkai Ling

**Affiliations:** ^1^ Department of Cardiology Zhongda Hospital Affiliated to Southeast University Nanjing China; ^2^ Department of Cardiology, School of Medicine Southeast University Nanjing China; ^3^ Department of Geriatrics Suzhou Ninth Hospital Affiliated to Soochow University Suzhou China; ^4^ Department of Radiation Oncology The First Affiliated Hospital of Nanjing Medical University Nanjing China

**Keywords:** cardiac fibrosis, m6A modification, METTL3, myocardial infarction, SMOC2

## Abstract

Cardiac fibrosis, especially in the infarct border zone, leads to decreased cardiac compliance, impaired systolic and diastolic function, resulting in heart failure. M6A methylation plays a role in fibrosis development. However, its underlying mechanism remains poorly understood. This study explores the role and molecular mechanisms of m6A methylation in regulating cardiac fibrosis after myocardial infarction (MI). A mouse myocardial fibrosis model post‐MI was established by ligating the left coronary artery. Corresponding gene knockdown was achieved in vitro or in vivo using short hairpin RNA or fibroblast‐specific AAV9 virus. Echocardiography assessed cardiac function in mice, while Masson staining determined the degree of collagen deposition post‐MI. The meRIP‐Seq kit detected mRNA methylation levels in myocardial tissue and hypoxia‐treated cardiac fibroblasts. Expression of RNA methylation‐related enzymes, fibrosis‐related proteins, and SMOC2 expression in the myocardial tissue or cardiac fibroblasts were detected using western blotting. Actinomycin D assessed SMOC2 mRNA stability. Results demonstrated increased levels of m6A methylation and METTL3 expression in myocardial fibrosis tissue post‐MI and in hypoxia‐treated cardiac fibroblasts. In vivo METTL3 downregulation reduced the fibrotic area and improved cardiac function, while METTL3 downregulation in vitro can alleviate cardiac fibroblast proliferation and differentiation after hypoxia. Mechanistically, METTL3 promoted SMOC2 mRNA stability by increasing its m6A methylation level, thereby regulating cardiac fibroblast proliferation and differentiation. Together, our work uncovers a critical link between METTL3 and SMOC2, providing insight into the functional importance of the mRNA m6A methylation and its modulators in cardiac fibrosis post MI.

## 1 Introduction

Heart failure (HF) is a significant public health issue affecting more than 23 million people worldwide [1]. With an aging population, its incidence has increased annually, imposing a huge medical burden on society [2, 3]. Among the numerous cardiovascular diseases, myocardial infarction (MI) is one of the main causes of HF. Necrotic myocardial cells are replaced by fibroblasts post‐MI, forming fibrotic scars that prevent ventricular wall rupture following ischaemic injury. Subsequently, owing to the increase in mechanical stress and activation of fibrogenic signalling pathways, fibroblasts in the infarct border zone further proliferate and differentiate, destroy the normal structure of myocardial tissue, reduce cardiac compliance, contribute to cardiac systolic and diastolic dysfunction, and ultimately lead to HF [3]. Therefore, clarifying the molecular mechanisms underlying the development of cardiac fibrosis in the infarct border zone post‐MI can help identify new intervention targets for preventing, stabilising, and even reversing HF and facilitate more precise clinical selection of anti‐HF treatment strategies.

Our previous studies showed that epigenetic modifications participate in the proliferation and differentiation of cardiac fibroblasts (CFs) [4, 5]. m6A modifications are reportedly associated with the development of myocardial fibrosis. Inhibition of FTO, an m6A demethylase, attenuates the antifibrotic effect of leonurine in rat cardiac fibroblasts [6]. METTL3 catalyses m6A methylation of GAS5 in a YTHDF2‐dependent manner to boost mitochondrial fission and cardiac fibroblast proliferation and fibroblast migration [7]. Overexpression of methyltransferase METTL3 in CFs could upregulate the expression of fibrosis‐related genes and activate the TGF‐β/Smad2/3 signalling pathway to promote the development of myocardial fibrosis [8]. METTL3 can also inhibit the expression of androgen receptors (AR) in an m6A‐YTHDF2‐dependent manner, thereby increasing cell glycolysis and promoting CF proliferation [9]. Despite these results, cardiac fibrosis remains a complex pathological process, necessitating further study of its specific molecular mechanisms to enhance the prognosis of heart failure.

SMOC2, a SPARC‐related modular calcium‐binding protein 2, is an encoded modular secretion protein that can affect the activity of cytokines, destroy the attachment of cell substrates, and regulate cell differentiation and the cell cycle [10, 11]. SMOC2 is reportedly involved in the regulation of fibrotic diseases [12, 13, 14]. Moreover, SMOC2 suppression has been shown to alleviate myocardial fibrosis via the ILK/p38 pathway [15]. Several analyses of expression profile data have identified SMOC2's potential involvement in the pathogenesis of HF, indirectly suggesting its association with cardiac fibrosis [16, 17]. However, the question of whether METTL3 promotes cardiac fibrosis by affecting the methylation level of SMOC2 remains unclear. Consequently, we undertook this study to investigate the effect of METTL3 on cardiac fibrosis and to understand its specific mechanism.

## 2 Materials and Methods

### 2.1 Animal Care and Treatments

Eight‐week‐old male C57BL/6 mice weighing 20–22 g were purchased from Qing Longshan Animal Breeding Field (Nanjing, China). All animal experiments were approved by the Ethics Review Board for Animal Studies of the Institute of Southeast University (Approval ID: 20230220020, Nanjing, China) and adhered to the established guidelines published by the US National Institutes of Health. Under standard environmental conditions, all animals were housed and had ad libitum access to food and water.

The mice were randomly divided into sham, 7 dpmi (7‐day post‐MI), 14 dpmi (14‐day post‐MI) and 28 dpmi (28‐day post‐MI) groups (*n* = 6 per group) and underwent surgical induction of MI by permanent coronary ligation or sham operation. Briefly, the mice were anaesthetised intraperitoneally with sodium pentobarbital (50 mg/kg, Merck, Germany). Following endotracheal intubation, they were placed on a ventilator, and a left lateral thoracotomy was performed to expose the left ventricle. The left coronary artery was ligated using an 8–0 silk suture. Successful ligation was confirmed by observing left ventricular pallor immediately after ligation. Mice in the sham group underwent similar surgery without ligation [4].

To investigate the role of METTL3 in vivo, AAV9 carrying a periostin promoter driving the expression of short hairpin RNA (shRNA) targeting METTL3 (AAV9‐periostin promoter‐eGFR‐shMETTL3, AAV9‐shMETTL3, TAATGTCCCATACGGTAGCTC) or a negative control (AAV9‐periostin promoter‐eGFR‐shNC, AAV9‐shNC, ACGTGACACGTTCGGAGAA) was constructed by Shanghai GeneChem Co. (Shanghai, China) [18, 19]. Briefly, the mice were randomly divided into four groups: AAV9‐shNC + sham (*n* = 6), AAV9‐shNC + MI (*n* = 6), AAV9‐shMETTL3 + sham (*n* = 6) and AAV9‐shMETTL3 + MI. At 3 days post MI or sham operation, each mouse was injected with AAV9‐shMETTL3 or AAV9‐shNC containing 2.5 × 10^11^ viral genome particles via the tail vein (Figure S1).

At 4 weeks post‐treatment, the animals were sacrificed after excessive inhalation of CO_2_ and their hearts were harvested.

### 2.2 Cell Culture and Treatment

CFs were isolated from freshly euthanized C57BL/6 mice through enzymatic digestion, following a standard protocol [20]. Briefly, mouse hearts were rapidly excised, minced, and placed in cold phosphate buffered saline (PBS). The minced tissue was subsequently digested with a collagenase II (#V900892, Sigma‐Aldrich, MO)/trypsin (Solarbio Life Sciences, Beijing) solution at 37°C for 8 min. After six to seven digestion periods, the supernatants were centrifuged to collect the cells. Cells were resuspended in DMEM/F12 (#11320033, Gibco, MA) and incubated for 90 min to allow CFs to attach to the dishes. Subsequently, the cells in the medium were discarded. CFs were incubated at 37°C in a humidified atmosphere of 5% CO_2_ and grown to 70%–80% confluence. CFs were further cultured and passaged at a 1:3 dilution. Second‐to fourth‐passage CFs were used in the experiment. In addition, for hypoxic experiments, the cells were incubated in 21% or 1% O_2_, for an additional 24, 48, 7 and 2 h [21].

### 2.3 Dot Blot Assay

RNA samples were isolated and purified using TRIzol reagent according to the standard protocol [8]. After quantification and denaturation (95°C, 5 min), mRNA was loaded onto Amersham HyBond *N*+ membranes (Amersham, UK). Subsequently, the membrane was crosslinked with UV light for 5 min, followed by staining with 0.02% Methylene blue. Photographs were taken to determine the input RNA content. Subsequently, the membranes were washed with PBST and then blocked with 5% defatted milk for 1 h, followed by incubation with an m6A antibody (1:1000, Epigentek, #A‐1801) at 4°C overnight. Dotted blots were visualised after incubation with secondary antibodies (1:10000, Proteintech, Cat No. SA00001‐2).

### 2.4 m6A RNA Methylation Quantification

An EpiQuik m6A RNA methylation quantification kit (Epigentek Group Inc., Farmingdale, NY, USA, P‐9005) was used to quantify N6‐methyladenosine RNA methylation. Briefly, 300 ng of RNA was added to the wells, followed by the addition of capture antibody solution. Then, the detection antibody solution was added to the assay wells according to the manufacturer's protocol. The m6A levels were quantified using colorimetry by reading the absorbance of each well at 450 nm [22].

### 2.5 Histology

The hearts were perfused with ice‐cold PBS to eliminate blood contamination and fixed in 4% paraformaldehyde overnight. Subsequently, they were embedded in paraffin and cut into sections of 5‐μm thickness [23]. Masson's trichrome staining was performed according to standard methods [4]. The collagen appears blue and the muscle appears red. Image analysis software ImageJ was used to obtain data on the stained area. The software automatically calculates the collagen area ratio, using the formula: collagen area/total tissue area × 100%.

### 2.6 MeRIP‐qPCR

The m6A immunoprecipitation (MeRIP) procedure was performed using the MeRIP m6A kit (17–10,499, Magna) according to the manufacturer's published instructions. Briefly, mRNA was fragmented and immunoprecipitated with Protein A beads pre‐incubated with the anti‐m6A antibody (ab208577, Abcam) in IP buffer. After elution, qPCR was performed to determine the mRNA levels. MeRIP‐qPCR was performed in triplicates [24]. The primer sequences used are listed as Table 1.

**TABLE 1:** The primer sequences used in MeRIP‐qPCR.

SMOC2	Forward primer	AGAGAGGTTTCTGGGGCG
	Reverse primer	CTCAGAAGTTCTCGGCGCT

### 2.7 Western Blot Analysis

Proteins were extracted using a Protein Extraction kit (KeyGEN Biotech, #KGB5303‐100), and their concentrations were determined using the BCA Protein Assay Kit (KeyGEN Biotech, #KGB2101‐1000). Subsequently, the proteins were separated by SDS‐PAGE and transferred onto PVDF membranes, which were then blocked with TBST containing 5% skimmed milk powder. Following blocking, the membranes were incubated overnight at 4°C with specific primary antibodies and subsequently detected using an ECL protocol with horseradish peroxidase‐conjugated IgG as the secondary antibody [4]. Primary and secondary antibodies were utilised according to the manufacturer's instructions (Table 2).

**TABLE 2:** Primary and secondary antibodies used in western blot and immunofluorescence.

Designation	Source or reference	Identifiers	Dilutions
METTL3 antibody	Proteintech	#15073–1‐AP	1:1000
ALKBH5 antibody	Proteintech	#16837–1‐AP	1:5000
FTO antibody	Proteintech	#27226–1‐AP	1:2000
ɑ‐SMA antibody	Proteintech	#14395–1‐AP	1:5000
METTL14 antibody	Proteintech	#26158–1‐AP	1:2000
HSP90 antibody	Proteintech	#13171–1‐AP	1:10000
Collagen I antibody	Proteintech	#67288–1‐Ig	1:10000
β‐Actin antibody	Proteintech	#66009–1‐Ig	1:50000
PCNA antibody	Proteintech	#10205–2‐AP	1:5000
SMOC2 antibody	CUSABIO	#CSB‐PA169309	1:500
GAPDH antibody	Proteintech	#60004–1‐Ig	1:100000
WTAP antibody	Proteintech	#60188–1‐Ig	1:10000
YTHDF2 antibody	Proteintech	#24744–1‐AP	1:10000
HRP‐conjugated Goat Anti‐Rabbit IgG (H + L)	Proteintech	#Cat No. SA00001‐2	1:10000
HRP‐conjugated Goat Anti‐Mouse IgG (H + L)	Proteintech	#Cat No. SA00001‐1	1:10000
DAPI staining agent	Servicebio	G1012	1:1000

### 2.8 Echocardiography

Echocardiography was performed using a Visual Sonics Vevo3100 small‐animal ultrasound scanner (FUJIFILM Visual Sonics Inc) to assess cardiac function. Mice were anaesthetised with 1.5% isoflurane and put in a supine position. Data from three consecutive cardiac cycles were analysed for each measurement. Left ventricular systolic dimension (LVDs), left ventricular diastolic dimension (LVDd), and the thickness of the septal and posterior wall thicknesses were measured. LV systolic volume (LV Vol s), LV diastolic volume (LV Vol d), LV ejection fraction (LVEF), and fractional shortening (FS) were calculated from these measurements.

### 2.9 Cell Transfection

Specific shRNAs against METTL3/SMOC2 and control shRNAs were synthesised by Shanghai GeneChem Co. (Shanghai, China). CFs were transfected with either shMETTL3/shSMOC2 or shRNA using Lipofectamine 3000 transfection reagent (Invitrogen, Carlsbad, USA) [4]. Relative shRNA sequences were as in Table 3.

**TABLE 3:** Relative shRNA sequences used in cell transfection.

sh‐METTL3#1	5′‐GCTGCACTTCAGACGAATTAT‐3′
sh‐METTL3#2	5′‐GGTCTGAACTCTTCAGCATCG‐3′
sh‐METTL3#3	5′‐GAAGACAAATCAACTGCAACG‐3′
sh‐SMOC2	5′‐GCUGAAAGUACGUCUAAUA‐3′
sh‐NC	5′‐TTCTCCGAACGTGTCACGT‐3′

### 2.10 Cell Counting Kit‐8 Assay

CFs were inoculated in 96 well plates and transfected with shNC or shMETTL3 under normoxic or hypoxic conditions for 48 h. Thereafter, 10 μL of CCK‐8 solution was added to each well and incubated for 2 h. The absorbance of each well was measured at 450 nm using a microplate reader (ELx800, Bio‐tech, Germany). Viability = (absorbance of sample)/ (absorbance of control).

### 2.11 5‐Ethynyl‐2′‐Deoxyuridine (EdU) Incorporation Assay

The EdU assay was procured from Donghuan (Shanghai, China). Initially, cells were treated with shNC, shMETTL3, or shSMOC2 under normoxic or hypoxic conditions for 48 h. Subsequently, the cells were incubated with 10 μM EdU solution for an additional 2 h. After fixation with 4% formaldehyde and the addition of 150 μL glycine, the cells were incubated with 0.5% Triton X‐100. Apollo reaction reagent (1×) was introduced, and then the cells were stained with 300 μL Hoechst (5 μg/mL) in darkness. EdU‐labelled and unlabeled cells were then counted under a microscope (TE2000‐U; Nikon), and pictures were taken [4].

### 2.12 Immunofluorescence of Tissue Sections

Paraffin‐embedded heart tissue sections were rehydrated in a series of graded ethanol solutions, followed by heating in ethylenediaminetetraacetic acid (EDTA) antigen retrieval buffer (ShareBio, SB‐MY001) at a low boiling point for 15 min. Subsequently, the sections were blocked with 1% BSA for 1 h. Primary antibodies against METTL3 and SMOC2 were incubated overnight, followed by a 2‐h incubation with secondary antibodies. Nuclear staining was performed using DAPI stain. Primary and secondary antibodies were utilised according to the manufacturer's instructions (Table 2). Images were captured using a Picture Scanner (Pannoramic MIDI, 3DHISTECH, Hungary) and analysed by lmage Auto Analysis System (C.V.2.4, Servicebio, China).

### 2.13 Bioinformatic Analysis

#### 2.13.1 scRNA‐Seq Analysis

The scRNA‐seq dataset GSE145154 of MI was retrieved from the GEO database (https://www.ncbi.nlm.nih.gov/), comprising scRNA‐seq data from left ventricle tissues of three infarcted myocardium and one healthy person. Large gene expression matrices were created using the “merge” function. Cells with > 500 genes, < 5000 genes, and < 20% mitochondrial genes were retained. Gene expression lists were normalised using the “Normalise Data” function and further scaled. The samples were integrated using the anchors method in the R package “Seurat”, and core cells were obtained by filtering scRNA‐seq. Principal component analysis (PCA) was performed on single‐cell samples, and 20 PCs were selected for UMAP algorithm analysis. Using the R package “Harmony,” 10 cell clusters were classified using the “FindClusters” function, and each cluster was manually annotated by the CellMarker database. Differential analysis was performed by using the “limma” package. *p*‐value < 0.05 and |Log2FC| > 1 were designated as differentially expressed genes (DEGs). The “ggplot2” package was used to show the localisation of genes.

#### 2.13.2 MeRIP‐Seq Analysis

The MeRIP‐seq dataset GSE131296 of MI was retrieved from the GEO database (https://www.ncbi.nlm.nih.gov/) and contained four ischemic group samples and 10 normal group samples. The R package “DESeq2” was used for differential analysis [25]; *p*‐value < 0.05 and |Log2FC| > 1 were designated as DEGs. The “pheatmap” package was used to depict the top DEGs. Then, GO and KEGG enrichment analyses were performed using the “clusterprofile” function.

#### 2.13.3 Protein‐Ligand Interaction Analysis

The METTL3 protein structure was obtained from the Uniprot database and SMOC2 mRNA was modelled using the Build and Edit Hucleic Acid module in BIOVIA Discovery Studio 2019 Client software (Dassault Systèmes, Paris, France). Protein‐nucleic acid docking was conducted through the HDOCK server (http://hdock.phys.hust.edu.cn/). The PLIP interaction analysis platform (https://plip‐tool.biotec.tu‐dresden.de/plip‐web/plip/index) was used to comprehensively describe and systematically analyse the binding sites.

### 2.14 mRNA Stability Analysis

Following transfection, CFs were incubated with actinomycin D (2 mg/mL; Sigma‐Aldrich) for 0, 3 and 6 h, and SMOC2 expression was examined using RT‐qPCR [26].

### 2.15 Statistical Analyses

Quantitative data were expressed as the mean ± standard deviation. All data were analysed using IBM SPSS Statistics for Windows version 26.0 (IBM Corp., Armonk, N.Y., USA). Student's t‐test was employed to determine the statistical significance between the two groups, while one‐way analysis of variance (ANOVA) was used for comparisons of data among multiple groups. Bioinformatic analyses were conducted using R language (Version 4.0.3). A significance level of *p* < 0.05 was regarded as a statistically significant difference, while *p* < 0.01 indicated a high statistically significant difference.

## 3 Results

### 3.1 Increased m6A and METTL3 in Fibrotic Tissue of Mice and Hypoxia‐Treated CFs

**FIGURE 1:**
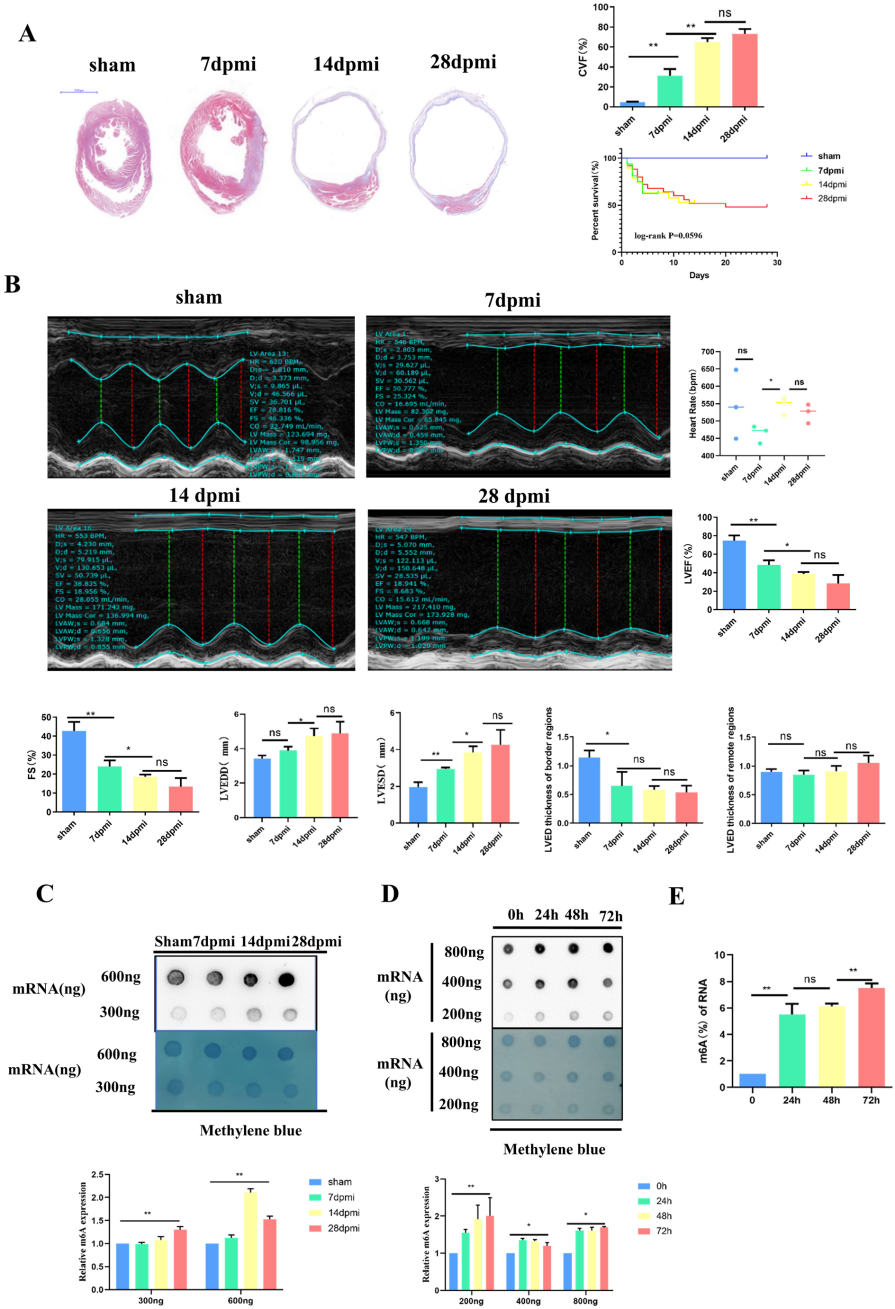
Increased m6A and METTL3 in fibrotic tissue of mice and Hypoxia‐treated CFs. (A) Masson staining and was used to analyse the degree of collagen deposition in the cardiac tissue of sham (*n* = 3), 7 dpmi (*n* = 3), 14 dpmi (*n* = 3), and 28 dpmi (*n* = 3) groups, and Kaplan–Meier analysis was used to analyse the survival rate of sham (*n* = 10), 7 dpmi (*n* = 16), 14 dpmi (*n* = 19), and 28 dpmi (*n* = 25) groups, log‐rank *p* = 0.0596; (B) Echocardiography was used to analyse of left ventricular ejection fraction and fractional shortening of sham (*n* = 3), 7 dpmi (*n* = 3), 14 dpmi (*n* = 3) and 28 dpmi (*n* = 3) groups, and; (C) Dot blot was used to detect the level of m6A of mRNA in left ventricular tissue of sham, 7dpmi, 14dpmi and 28dpmi groups; (D and E) Dot blot and m6A RNA methylation quantification were used to detect the levels of m6A of mRNA in CFs under hypoxia conditions at 0, 24, 48 and 72 h; *Indicating significant statistical differences: *p < 0.05 and **p < 0.01; 7 dpmi, 7 days after myocardial infarction; 14 dpmi, 14 days after myocardial infarction; 28 dpmi, 28 days after myocardial infarction; CVF, collagen volume fraction; FS, Fractional shortening; LVED, left ventricular end diastolic; LVEDD, left ventricular end diastolic diameter; LVEF, left ventricular ejection fraction; LVESD, left ventricular end systolic diameter; Sham, Sham surgery group.

**FIGURE 2:**
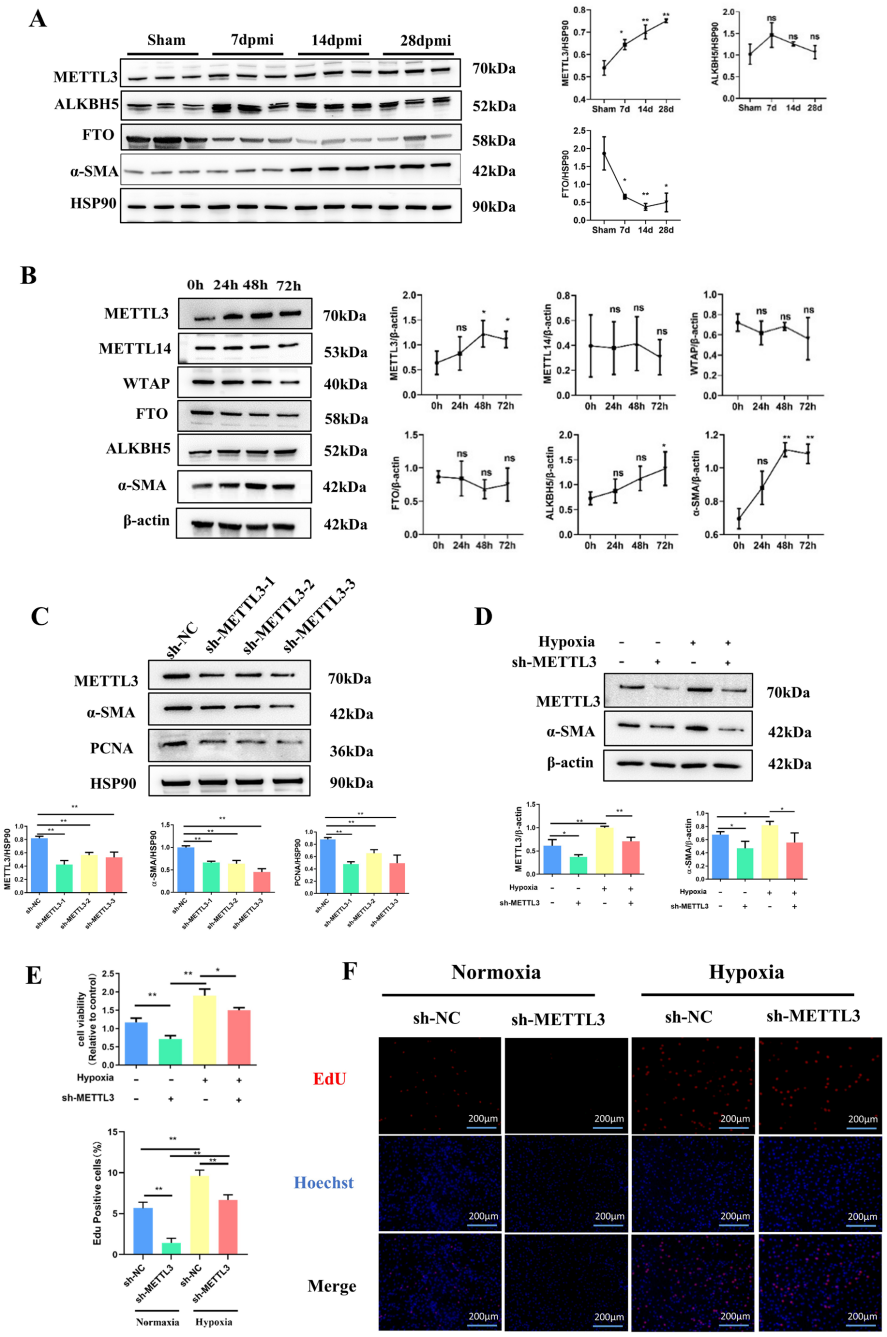
METTL3‐Mediated m6A modification regulates the proliferation and differentiation process of cardiac fibroblasts and the progression of cardiac fibrosis post‐MI. (A) Western blot was used to analyse the expression of METTL3, ALKBH5, FTO, ɑ‐SMA, HSP90 in left ventricular tissue of sham, 7 dpmi, 14 dpmi, and 28 dpmi groups; (B) Western blot was used to analyse the expression of METTL3, METTL14, WTAP, ALKBH5, FTO, ɑ‐ SMA, β‐Actin in CFs under hypoxia conditions at 0, 24, 48 and 72 h; (C) Western blot was used to analyse the expression of METTL3, ɑ‐SMA, PCNA and HSP90 in CFs with the treatment of sh‐NC or sh‐METTL3; (D) Western blot was used to analyse the expression of METTL3, ɑ‐SMA and HSP90 in CFs after treating with shNC or shMETTL3 under normoxic or hypoxic conditions; (E and F) CCK‐8 and EdU assay were used to detect the proliferation degree of CFs after treating with shNC or shMETTL3 under normoxic or hypoxic conditions; *Indicating significant statistical differences: *p < 0.05 and **p < 0.01; 7 dpmi, 7 days after myocardial infarction; 14 dpmi, 14 days after myocardial infarction; 28 dpmi, 28 days after myocardial infarction; Sham, Sham surgery group.

A cardiac fibrosis mouse model was established by ligating the left anterior descending coronary artery for 4 weeks. Masson's trichrome staining revealed an increase in cardiac collagen deposition (Figure 1A) accompanied by a deterioration in LVEF and FS post‐MI. The LVESD and LVEDD were both significantly increased at 14 dpmi and 28 dpmi compared to 7 dpmi and sham groups. This discrepancy was consistent with the enlargement of the left ventricle observed at 14 and 28 dpmi in heart slices. The LV end‐diastolic thickness of border regions was reduced post MI compared with the sham group, but no statistically significant difference was observed among the groups post MI (Figure 1B). Furthermore, we quantified m6A levels in RNA extracted from fibrotic tissues and hypoxia‐treated CFs, comparing them with m6A levels in sham surgical controls and normoxia‐treated CFs. The efficiency of hypoxia in vitro and in vivo was tested by measuring the expression of HIF‐1alpha (Figure S2). The increase in m6A in total RNA was observed as early as 7 days post‐MI in mice (Figure 1C). We also observed a sustained increase in the m6A levels after 24, 48 and 72 h of hypoxia (Figure 1D,E). To identify the regulators of elevated m6A levels in fibrotic tissues and hypoxia‐treated CFs, we measured the expression of several known proteins associated with m6A methylation. Western blotting demonstrated that the protein expression level of METTL3 was significantly increased in fibrotic tissues and hypoxia‐treated CFs (Figure 2A,B). Collectively, these data establish that METTL3 could be an important molecular hallmark that may regulate myocardial fibrosis post‐MI by increasing m6A methylation.

### 3.2 METTL3‐Mediated m6A Modification Regulates the Proliferation and Differentiation Process of Cardiac Fibroblasts and the Progression of Cardiac Fibrosis Post‐MI

**FIGURE 3:**
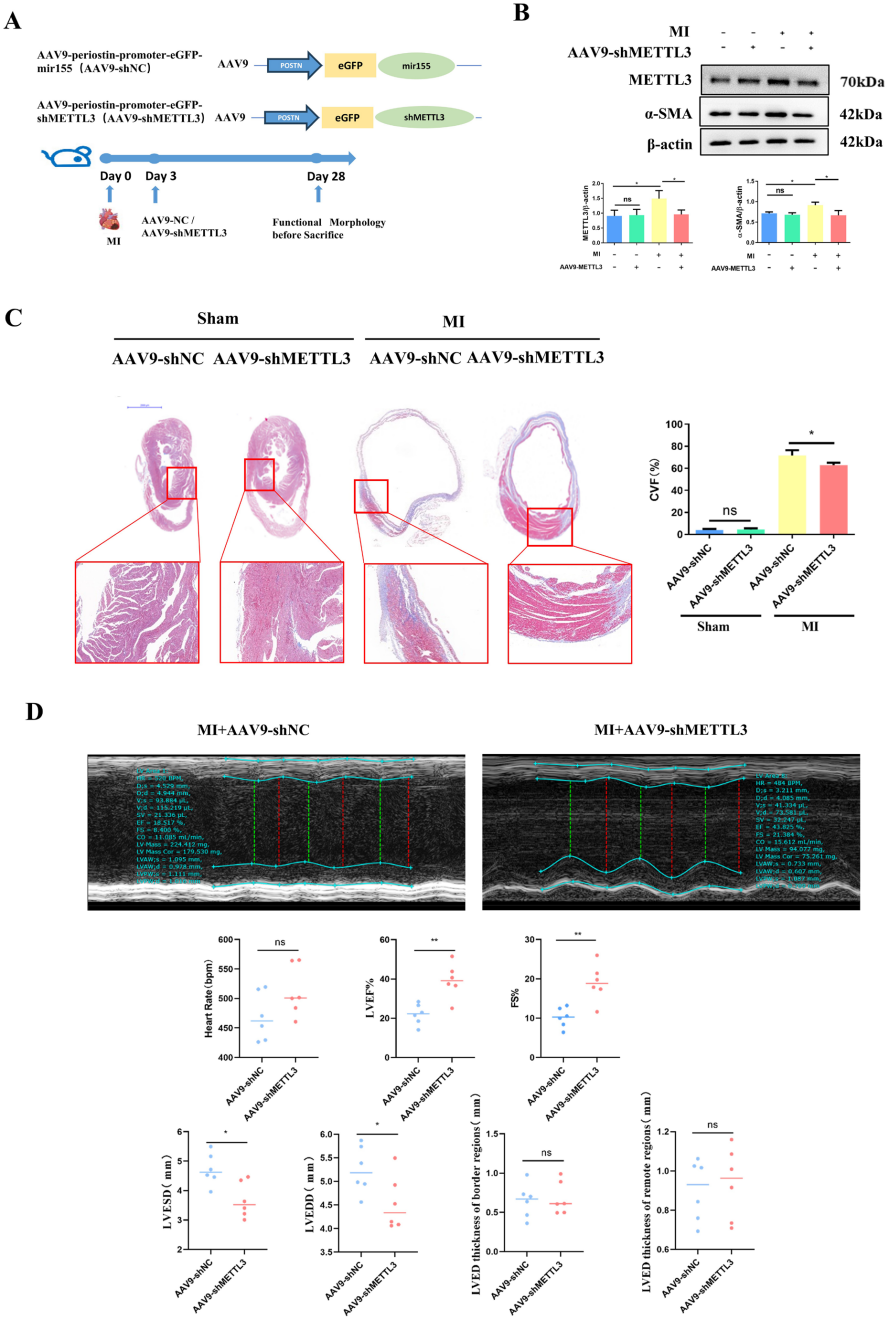
METTL3‐Mediated m6A modification regulates the proliferation and differentiation process of cardiac fibroblasts and the progression of cardiac fibrosis post‐MI. (A) Construct AAV9‐periostin promoter‐eGFR‐shMETTL3 and AAV9‐periostin promoter‐eGFR‐shNC, each mouse was injected with AAV9‐shMETTL3 or AAV9‐shNC via tail vein at 3 days post MI or sham operation; (B) Western blot was used to analyse the expression of METTL3, ɑ‐SMA and β‐Actin in left ventricular tissue with the treatment of AAV9‐METTL3 or AAV9‐shNC under MI or sham operation; (C) Masson staining was used to analyse the degree of collagen deposition in left ventricular tissue with the treatment of AAV9‐METTL3 or AAV9‐shNC under MI or sham operation (Sham+AAV9‐shNC *n* = 3; Sham+AAV9‐shMETTL3 *n* = 3; MI + AAV9‐shNC *n* = 3; MI + AAV9‐shMETTL3 *n* = 3); (D) Echocardiography was used to analyse of LVEF% and FS% in mice with the treatment of AAV9‐METTL3 (*n* = 6) or AAV9‐shNC (*n* = 6) under MI; CVF, collagen volume fraction; FS, fractional shortening; LVED, left ventricular end diastolic; LVEDD, left ventricular end diastolic diameter; LVEF, left ventricular ejection fraction; LVESD, left ventricular end systolic diameter. *Indicating significant statistical differences: *p < 0.05 and **p < 0.01.

To investigate whether METTL3 regulates the proliferation and differentiation of cardiac fibroblasts (CFs), we constructed sh‐METTL3 to knock down METTL3 expression (Figure 2C). All three shRNAs demonstrated high knockdown efficiency, and we selected sh‐METTL3‐1 for subsequent studies. CFs were treated with hypoxia and transfected with sh‐METTL3 for 48 h. We found that METTL3 downregulation significantly abrogated the hypoxia‐induced increase in α‐SMA expression (Figure 2D). Meanwhile, by using the CCK‐8 assay, we found that downregulation of METTL3 significantly abrogated the hypoxia‐induced proliferation (Figure 2E). The EdU incorporation assay results were consistent with the CCK‐8 assay (Figure 2F). In vivo, we constructed AAV9‐periostin promote‐eGFP‐shMETTL3 (AAV9‐shMETTL3), which specifically knocked down METTL3 in fibroblasts (Figure 3A). The transfection efficiency of the AAV9 virus in myocardial tissue was detected at 28 dpmi, and the AAV9 virus was transfected successfully into myocardial tissue (Figure S1), especially into cardiac myofibroblasts (Figure S3). Three days post‐MI, AAV9‐shMETTL3 was injected into the tail vein of mice (Figure 3A). Four weeks after the operation, AAV9‐shMETTL3 directionally knocked down the expression of METTL3 in the ischemic myocardium group without affecting the normal group. Considering that the acute phase of myocardial infarction had passed, this also explains why we did not observe more mice dying from cardiac rupture post‐MI after injection. We also found that the expression of ɑ‐SMA (Figure 3B) and PCNA (Figure S4) was decreased upon knockdown of METTL3. Moreover, the collagen deposition area in the infarction border zone was reduced (Figure 3C), and heart function was improved, which was manifested by the increased LVEF and FS and decreased LVESD and LVEDD (Figure 3D) in the MI + AAV9‐shMETTL3 group compared to the MI + AAV9‐sh‐NC group. These results indicated that METTL3 regulates the proliferation and differentiation of CFs and the progression of cardiac fibrosis post‐MI.

### 3.3 SMOC2 May Be a Key Gene Regulated by RNA Methylation During the Proliferation and Differentiation of Cardiac Fibroblasts

**FIGURE 4:**
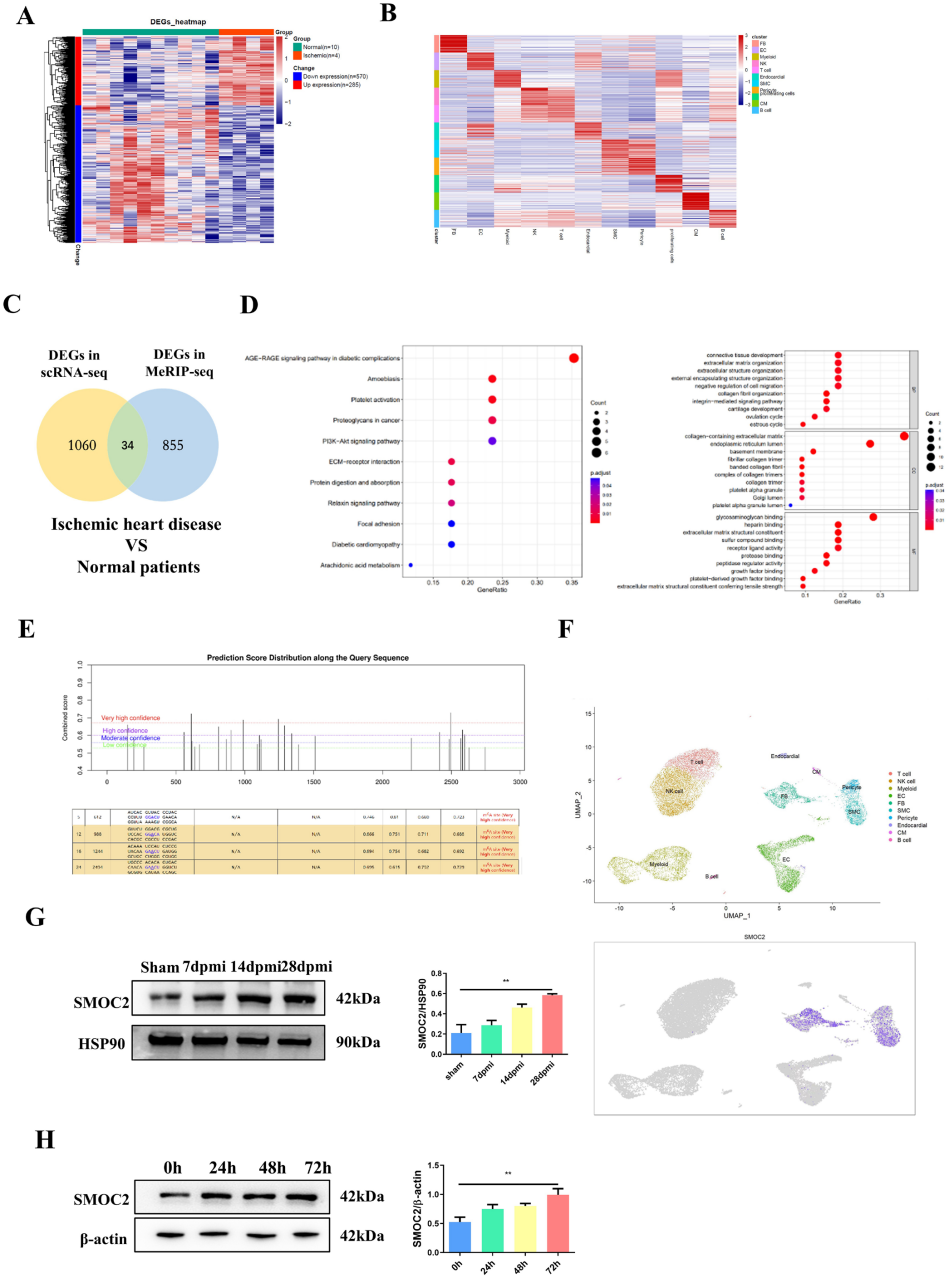
SMOC2 May be a key gene regulated by RNA methylation during the proliferation and differentiation of cardiac fibroblasts. (A) Comparison of differentially expressed genes in myocardial tissue RNA methylation sequencing results between patients with ischemic cardiomyopathy and normal individuals through heatmap analysis; (B) Cluster analysis of single‐cell sequencing results of myocardial tissue from patients with ischemic cardiomyopathy and normal individuals using UMP; (C) The Venn diagram displays the same differentially expressed genes in RNA methylation sequencing and single‐cell sequencing of myocardial tissue between patients with ischemic cardiomyopathy and normal individuals; (D) KEGG analysis reveals the signalling pathways that differentially expressed genes may participate in; GO analysis showcases the potential functions of differentially expressed genes; (E) The methylation sites of METTL3 on SMOC2 were predicted using the SRAMP database; (F) Single‐cell sequencing showcases the expression of SMOC2 in fibroblast clusters; (G) Western blot was used to analyse the expression of SMOC2 and HSP90 in left ventricular tissue of sham, 7 dpmi, 14 dpmi, and 28 dpmi groups; (H) Western blot was used to analyse the expression of SMOC2 and β‐Actin in CFs under hypoxia conditions at 0, 24, 48 and 72 h; 7 dpmi, 7 days after myocardial infarction; 14 dpmi, 14 days after myocardial infarction; 28 dpmi, 28 days after myocardial infarction; Sham, Sham surgery group.*Indicating significant statistical differences: *p < 0.05 and **p < 0.01.

To further reveal the mechanism of CFs proliferation and differentiation after myocardial infarction, we utilised the GEO database (https://www.ncbi.nlm.nih.gov/geo/) to retrieve the single‐cell RNA Sequencing (scRNA‐Seq) dataset GSE145154 and the Methylated RNA Immunoprecipitation Sequencing (MeRIP‐Seq) dataset GSE131296. Thereafter, we performed a secondary analysis on the gene expression profiles of normal humans and patients with ischaemic cardiomyopathy heart tissues. We then used Log FC > 1 and *p* < 0.05 as the screening conditions to obtain differential genes (Figure 4A,B). Thirty‐four differentially expressed genes (DEGs) were identified when we combined the two aforementioned differential genomes (Figure 4C). Subsequently, we conducted GO and KEGG analyses using these 34 differential genes to further investigate their potential functions and affected pathways (Figure 4D). Further analysis showed that SMOC2, one of the 34 differentially expressed genes, possessed multiple highly confident m6A methylation sites (Figure 4E) and exhibited relatively high expression abundance in the FB cluster (Figure 4F).

**FIGURE 5:**
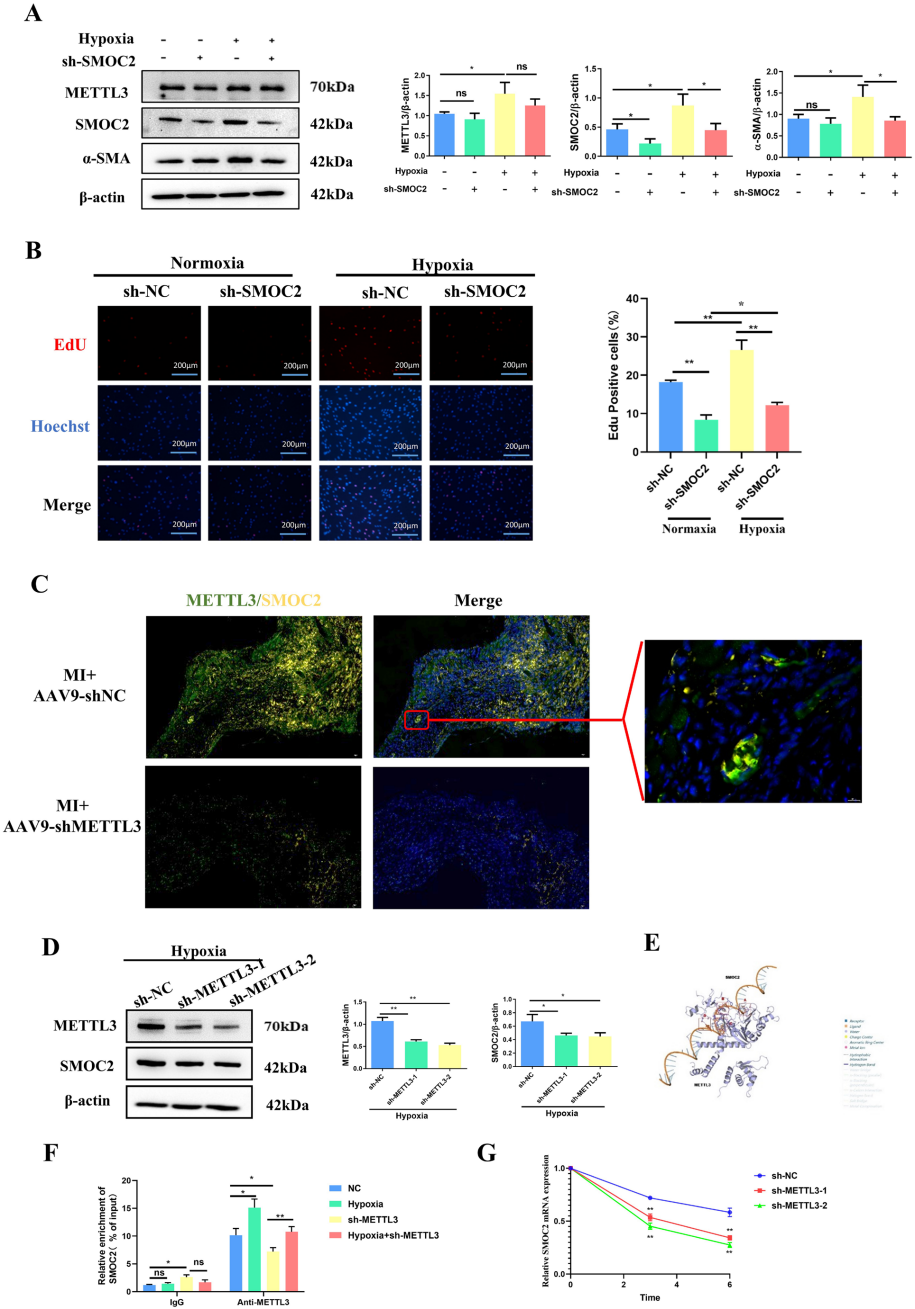
METTL3 regulates SMOC2 expression by increasing the stability of SMOC2 mRNA. (A) Western blot was used to analyse the expression of METTL3, SMOC2, ɑ‐SMA, and GAPDH in CFs after treatment with shNC or shSMOC2 under normoxic or hypoxic conditions; (B) EdU assay was used to detect the proliferation degree of CFs after treatment with shNC or shSMOC2 under normoxic or hypoxic conditions. (C) Immunofluorescence was used to detect METTL3 and SMOC2 expression in border zone of left ventricular tissue with treatment of AAV9‐METTL3 (*n* = 3) or AAV9‐shNC (*n* = 3) under MI operation; (D) Western blot was used to analyse the expression of METTL3, SMOC2, ɑ‐SMA, and GAPDH in CFs with the treatment of shNC, shMETTL3‐1 or shMETTL3‐2 under hypoxic conditions; (E) The interaction core sites of METTL3 and SMOC2 were predicted using PLIP; (F) MeRIP‐qPCR was used to detect SMOC2 mRNA expression in CFs following treatment with shMETTL3 or shNC under normoxic or hypoxic condition; (G) SMOC2 stability was evaluated using RT‐qPCR following treatment with actinomycin D. *Indicating significant statistical differences: *p < 0.05 and **p < 0.01.

To further clarify the role of SMOC2 in the proliferation and differentiation of CFs, we used western blotting to detect the expression levels of SMOC2 in mouse myocardial fibrosis tissues and hypoxic intervention in CFs after MI. Results showed that the expression level of SMOC2 gradually increased with prolonged ischaemia and hypoxia time in myocardial fibrosis tissues and hypoxic intervention in CFs after MI (Figure 4G,H). Knockdown of SMOC2 in CFs did not affect the expression of METTL3 but regulated the proliferation and differentiation of CFs (Figure 5A,B). However, regulating the expression of METTL3 in a myocardial fibrosis model after MI or hypoxia intervention in CFs can affect the expression level of SMOC2 (Figure 5C,D); therefore, we initially believe that METTL3 can affect the proliferation and differentiation of CFs by regulating the expression level of SMOC2.

### 3.4 METTL3 Regulates SMOC2 Expression by Increasing the Stability of SMOC2 mRNA

To elucidate the mechanism by which METTL3 regulates SMOC2 expression, we examined the effects of METTL3 on SMOC2 methylation. As shown in Figure 4E, multiple methylation sites were predicted. Among these, four sequences were the most likely binding sites (Figure 4E). PLIP was used to analyse the interaction core sites between METTL3 and SMOC2 mRNA. According to the results shown in Figure 5E, binding sites exist between METTL3 and SMOC2 mRNA. We conducted MeRIP‐qPCR experiments and observed that METTL3 affected the binding of m6A to SMOC2 mRNA (Figure 5F). Actinomycin D experiments showed that the METTL3 knockdown reduced the stability of SMOC2 mRNA (Figure 5G). Therefore, we considered that METTL3 regulated SMOC2 expression by affecting the stability of SMOC2 mRNA, thereby affecting the proliferation and differentiation of cardiac fibroblasts.

## 4 Discussion

M6A methylation modification may be a potential target for the treatment of heart failure [27, 28]. The proliferation and differentiation of cardiac fibroblasts in the infarct border zone are key pathological processes in the progression of heart failure [29, 30, 31]. Studies have indicated the involvement of m6A methylation modification in the proliferation and differentiation of cardiac fibroblasts [32, 33, 34], a finding corroborated by our study, which observed increased m6A methylation in a model of hypoxia‐induced cardiac fibroblast proliferation and differentiation, as well as cardiac fibrosis post‐MI. Moreover, the level of m6A methylation increased with the extension of hypoxia and ischaemia time. Three types of molecules are implicated in the modification of m6A: the m6A methyltransferase complex (METTL3, METTL14, and WTAP complex), RNA demethylase FTO and ALKBH5, and binding proteins containing the YTH domain [35, 36]. However, controversy persists regarding the enzymes that play major roles in the proliferation and differentiation of cardiac fibroblasts. METTL3, WTAP [37] and FTO [6] reportedly regulate CF proliferation and differentiation. Our study found that in cardiac fibrotic tissue post‐MI, the expression of METTL3 and ALKBH5 increased, whereas that of FTO decreased with the extension of ischaemia time. However, in CFs treated with hypoxia, the expression of METTL3 and ALKBH5 was upregulated with the extension of hypoxia time, whereas the expression of FTO and other m6A relative enzymes was not significantly different. Combined with the results showing that m6A methylation was increased in CFs treated with hypoxia and in cardiac fibrotic tissue post‐MI, we finally chose to study the role of METTL3 in the process of myocardial fibrosis after myocardial infarction. Further, our research found that knockdown of METTL3 in vivo and in vitro can reduce the expression of ɑ‐SMA, inhibit the proliferation of CFs, reduce the area of cardiac fibrosis post‐MI and improve cardiac function. So we believe that METTL3 plays a key role in regulating the proliferation and differentiation of CFs via m6A methylation. Currently, studies have indicated that during the process of cardiomyocyte apoptosis induced by hypoxia‐reoxygenation, the elevated expression of METTL3 modulates the methylation of the downstream TFEB gene, leading to its upregulated expression. This, in turn, decreases cardiomyocyte autophagy and enhances apoptosis. However, increasing TFEB expression can enhance ALKBH5 expression while suppressing METTL3 expression, forming a compensatory mechanism that regulates cardiomyocyte apoptosis [38]. Another study exploring the role of RNA methylation in osteogenic differentiation revealed that upregulating METTL3 inhibits osteogenic processes, yet this inhibition can be reversed by ALKBH5, further underscoring the existence of a compensatory mechanism between METTL3 and ALKBH5, which allows for dynamic reversibility in RNA methylation [39]. Our study also observed dynamic changes in METTL3 and ALKBH5 expression during myocardial fibrosis post‐myocardial infarction and in the proliferation and differentiation of hypoxic‐induced cardiac fibroblasts, suggesting that a compensatory mechanism involving METTL3 and ALKBH5 may also exist in the proliferation and differentiation of cardiac fibroblasts, albeit this hypothesis warrants further experimental validation in the future.

METTL3 mainly regulates the expression of target genes by affecting m6A methylation levels through post‐transcriptional modification [32]. METTL3 also regulates m6A levels and the expression of collagen‐related genes [8], IGFBP3, androgen receptors [9], and lncRNA GAS5, all of which affect the proliferation and differentiation of CFs. With advances in next‐generation sequencing technology, an increasing number of genes have been thoroughly explored. In this study, we conducted a secondary analysis of scRNA‐seq and MeRIP‐seq data from myocardial ischemic tissue samples obtained from the GEO database. Through this analysis, we identified 34 DEGs regulated by m6A and associated with myocardial fibrosis. Among these, the role of SMOC2 piqued our interest. Increasing preclinical studies have demonstrated that SMOC2 is involved in regulating fibrotic diseases [14, 40, 41]. Research has shown that in renal fibrosis, SMOC2 contributes to fibrogenesis by enhancing inflammatory responses and activating the MAPK, SMAD and AKT signalling pathways. Additionally, SMOC2 promotes pulmonary fibrosis and hepatic steatosis by modulating the TGF‐β/SMAD, AKT and ERK signalling pathways [10, 13, 42]. In myocardial fibrosis, SMOC2 participates in regulating the proliferation and differentiation of cardiac fibroblasts through activation of the ILK/p38 signalling pathway [15]. However, our study on the role of SMOC2 in myocardial fibrosis is limited. Our study confirmed that SMOC2 was upregulated in myocardial fibrosis post‐MI and in CFs treated under hypoxic conditions and that knockdown of SMOC2 could regulate the proliferation and differentiation of CFs. Up to now, no studies have revealed the upstream regulatory mechanisms of SMOC2 in the proliferation and differentiation of CFs. Our study is the first to show that METTL3 affects the degradation rate of SMOC2 mRNA by modulating SMOC2 mRNA m6A methylation levels, thereby regulating SMOC2 expression. Together, our work uncovers a critical link between METTL3 and SMOC2, providing insight into the functional importance of the mRNA m6A methylation and its modulators in cardiac fibrosis post MI.

However, the present study had several limitations. Firstly, owing to difficulty in collecting human left ventricular samples, our research performed analyses based on data in an online public database. Secondly, while in the present study we verified that SMOC2 can affect the proliferation and differentiation of CFs, its potential mechanism is not clear. Therefore, further research is necessary to unravel the downstream mechanism of SMOC2 in the proliferation and differentiation of CFs. Thirdly, the research was conducted exclusively using male mice, which may limit the generalisability of the findings to female populations. The absence of female mice in this study prevents an assessment of whether the observed effects are consistent across sexes. Future studies should include both male and female mice to ensure a more comprehensive understanding of the outcomes and their potential applicability to all individuals.

## 5 Conclusions

**FIGURE 6:**
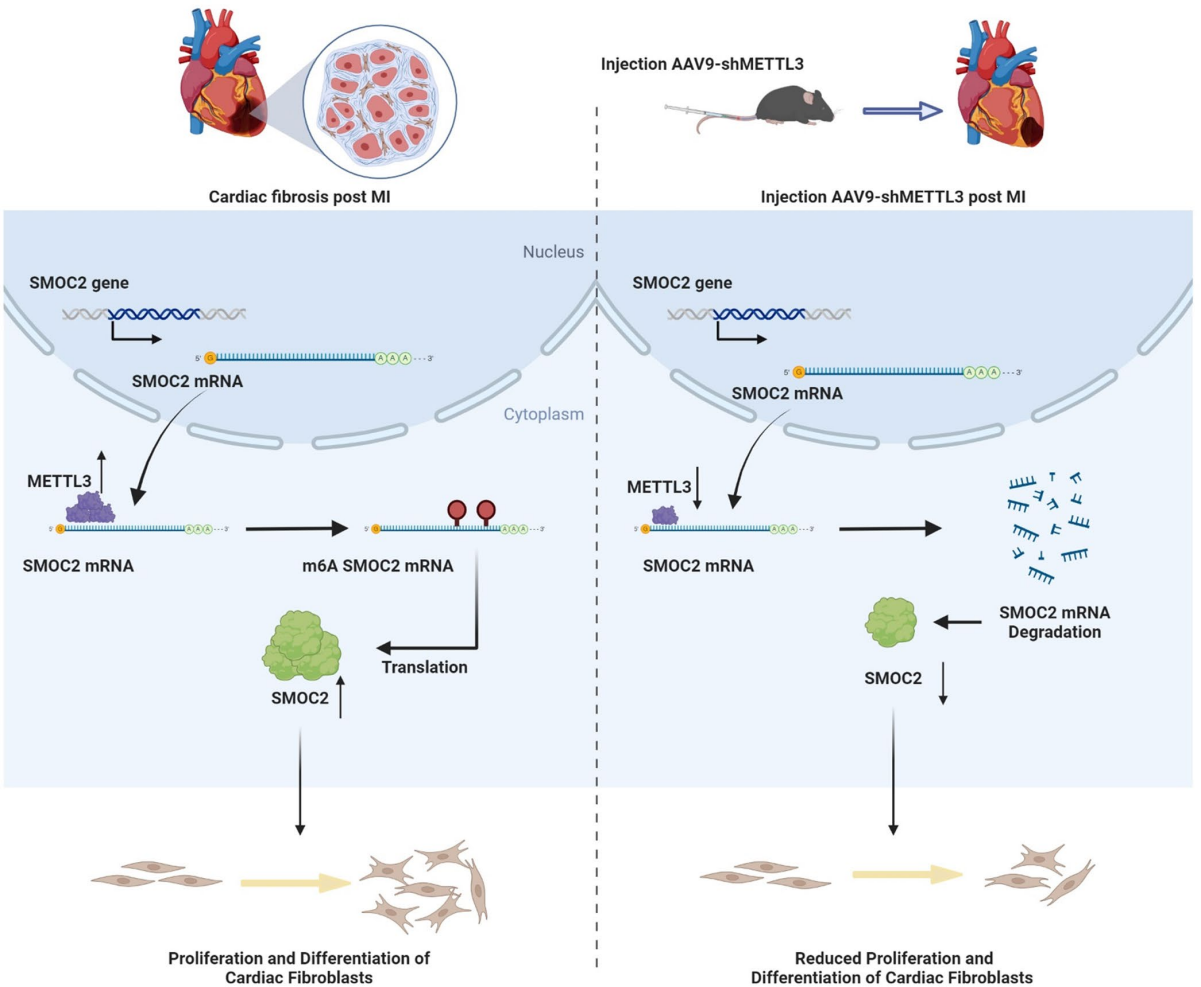
Mechanistic scheme. (A) METTL3 is increased post MI, and METTL3 promotes SMOC2 mRNA stability by increasing m6A methylation level, thereby participating in CF proliferation and differentiation. (B) Downregulation of METTL3 decreases the SMOC2 mRNA stability by decreasing m6A methylation level, subsequently curtailing the CF proliferation and differentiation.

According to the results of the present study, METTL3 expression is increased in cardiac fibrosis post MI. We propose that METTL3 promotes SMOC2 mRNA stability by increasing the m6A methylation level, thereby participating in CF proliferation and differentiation after MI (Figure 6). Our results could provide new targets for application in the treatment of cardiac fibrosis.

## Author Contributions

**Yanru He:** funding acquisition (equal), writing – original draft (equal). **Xiaodong Pan:** data curation (equal), investigation (equal). **Zhuyuan Liu:** writing – review and editing (equal). **Pengfei Zuo:** formal analysis (equal). **Zulong Sheng:** formal analysis (equal). **Chunshu Hao:** writing – review and editing (equal). **Zaixiao Tao:** data curation (equal), formal analysis (equal). **Zhongpu Chen:** resources (equal). **Jiali Song:** formal analysis (equal). **Genshan Ma:** conceptualization (equal). **Sunkai Ling:** investigation (equal), methodology (equal).

## Ethics Statement

Animal Studies: Experiments using animals have been approved by Animal Experimental Ethical Inspection Form of Southeast University, No. 20230220020.

## Consent

The authors have nothing to report.

## Conflicts of Interest

The authors declare no conflicts of interest.

## Supporting information


**Figures S1–S4:** jcmm70829‐sup‐0001‐FigureS1‐S4.docx.

## Data Availability

All data, models or code generated or used during the study are available from the corresponding author by request.
