# Supplementary material for: METTL3 Silencing Suppresses Cardiac Fibrosis Post Myocardial Infarction via m6A Modification of SMOC2
Source: J Cell Mol Med. 2025 Sep 5;29(17):e70829. doi: 10.1111/jcmm.70829 (PMC12413310; doi:10.1111/jcmm.70829)
Supplement: Supplementary file 1 — Figures S1–S4: jcmm70829‐sup‐0001‐FigureS1‐S4.docx. [file JCMM-29-e70829-s001.docx]

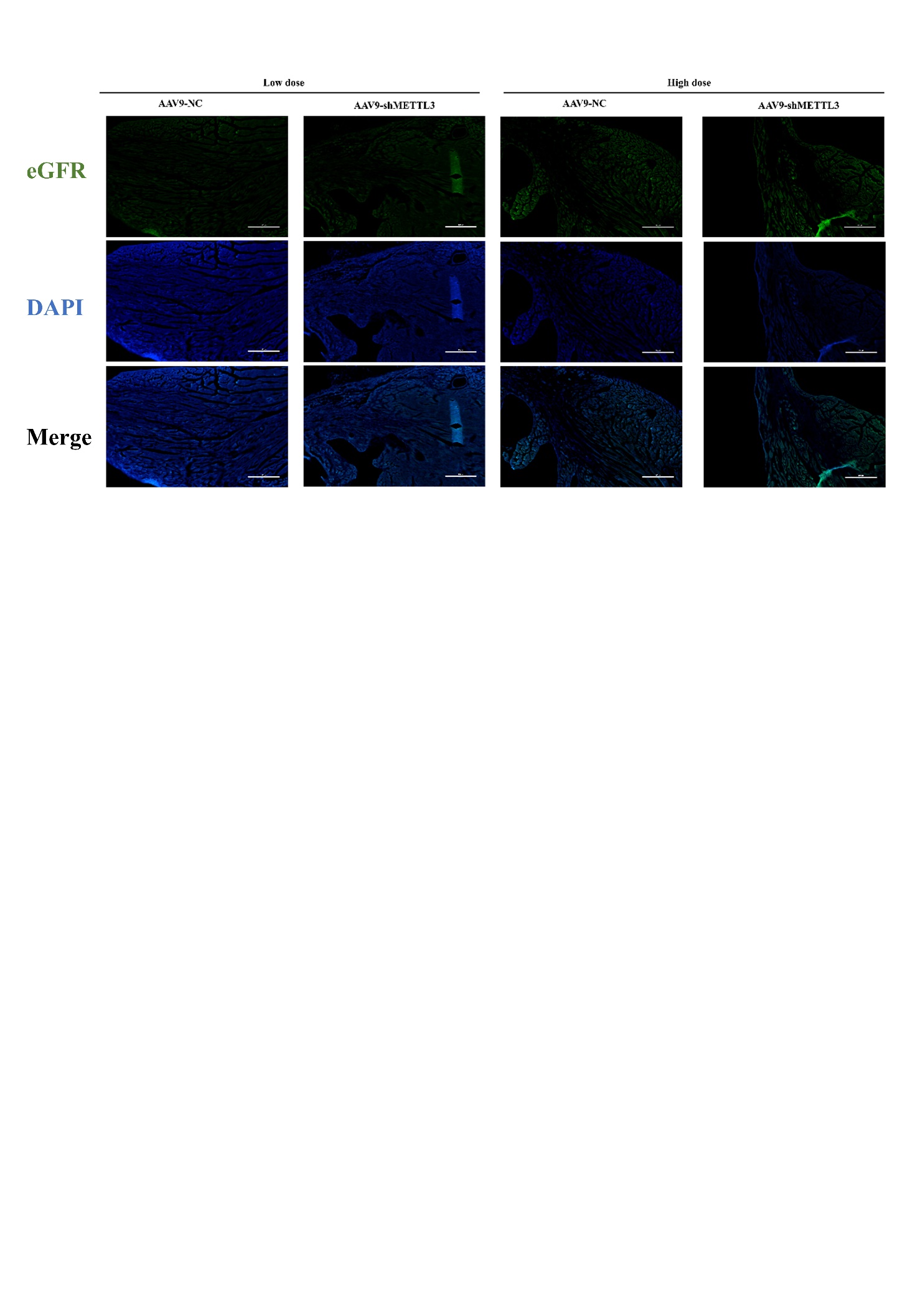


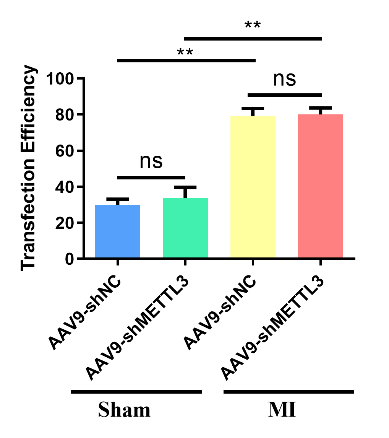


**Supplementary Figure 1: Transfection efficiency of cardiac fibroblasts after intervention with different concentrations of AAV9-shNC or AAV9-shMETTL3 under sham or MI operation. Immunofluorescence showed the expression of EGFR.**


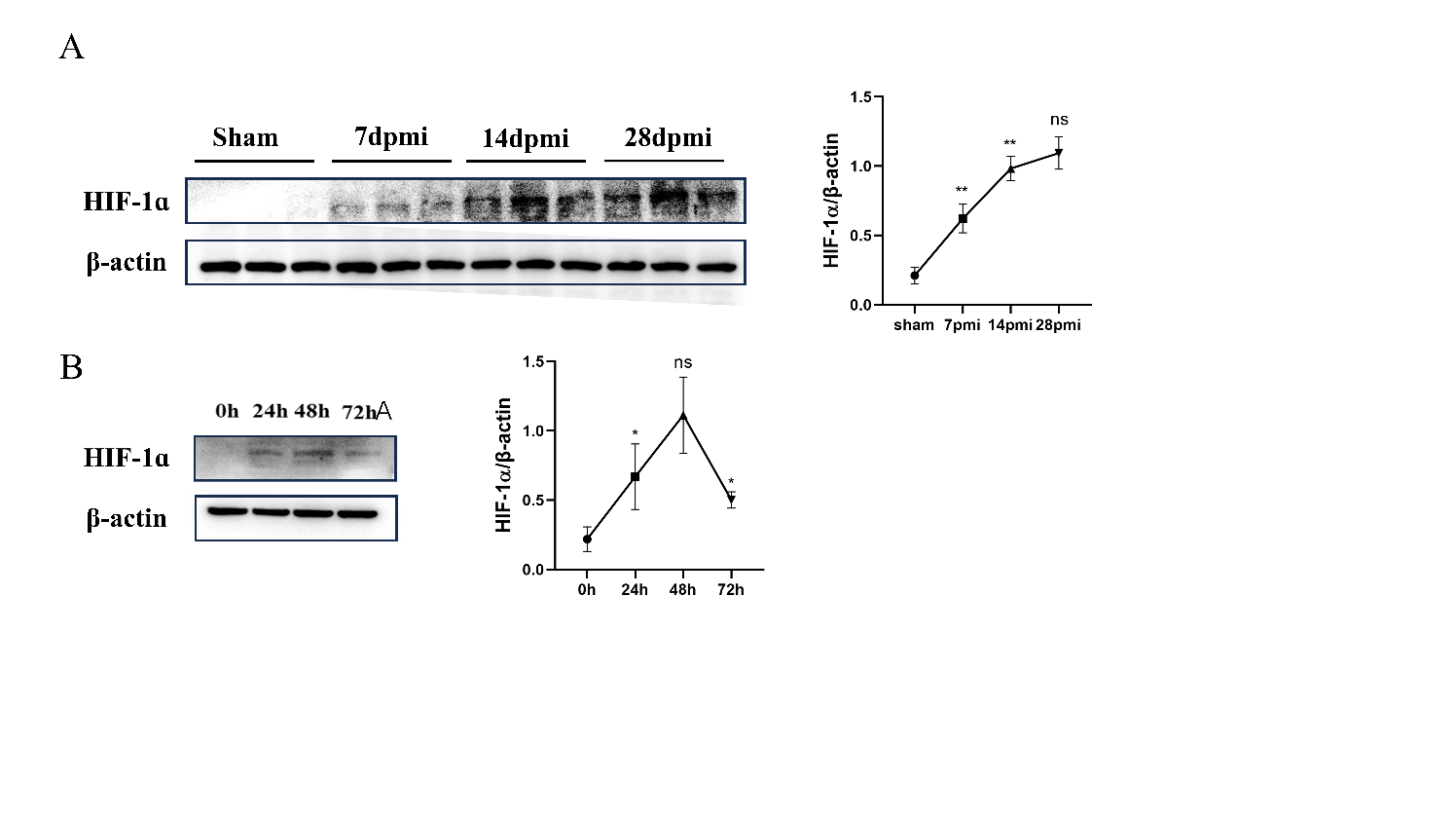


**Supplementary Figure 2: A) Western blot was used to analyze the expression of HIF-1ɑ, β-actin in left ventricular tissue of sham, 7 dpmi, 14 dpmi, and 28 dpmi groups; B) Western blot was used to analyze the expression of HIF-1ɑ, β-actin in CFs under hypoxia conditions at 0, 24, 48 and 72 h.**


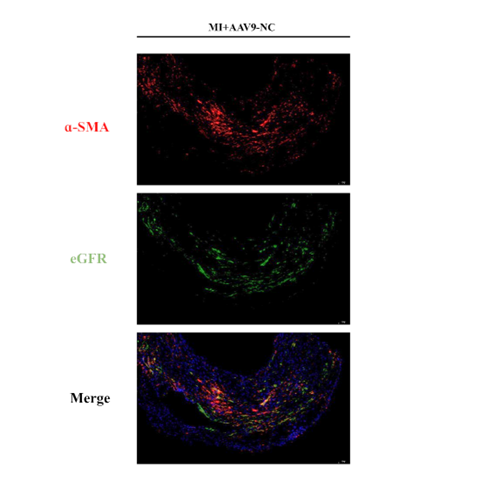


**Supplementary Figure 3: Immunofluorescence showed the expression and localization of a-SMA and eGFR after intervention with AAV9-shNC post myocardial infarction.**


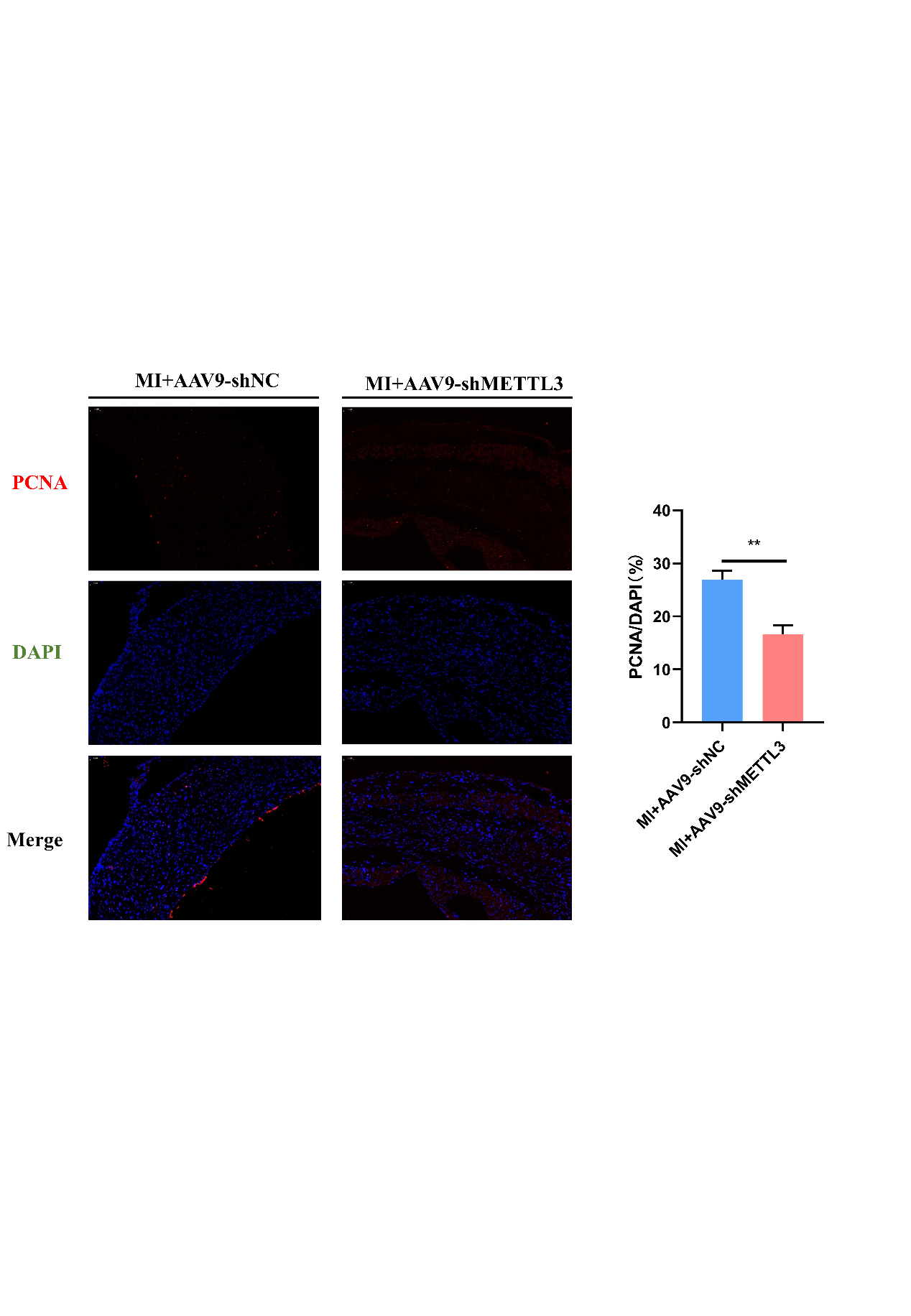


**Supplementary Figure 4: Immunofluorescence was used to detect PCNA expression in border zone of left ventricular tissue with treatment of AAV9-METTL3 (n=3) or AAV9-shNC (n=3) under MI operation.**
